# Active Multimodal Sensor System for Target Recognition and Tracking

**DOI:** 10.3390/s17071518

**Published:** 2017-06-28

**Authors:** Yufu Qu, Guirong Zhang, Zhaofan Zou, Ziyue Liu, Jiansen Mao

**Affiliations:** School of Instrumentation Scienc & Optoelectronics Engineering, Beihang University, Beijing 100191, China; gr_zhang@buaa.edu.cn (G.Z.); zouzhaofan@buaa.edu.cn (Z.Z.); liuziyue10171073@buaa.edu.cn (Z.L.); mjs110333@buaa.edu.cn (J.M.)

**Keywords:** multisensor, active collection, sensor cueing, target recognition, target tracking

## Abstract

High accuracy target recognition and tracking systems using a single sensor or a passive multisensor set are susceptible to external interferences and exhibit environmental dependencies. These difficulties stem mainly from limitations to the available imaging frequency bands, and a general lack of coherent diversity of the available target-related data. This paper proposes an active multimodal sensor system for target recognition and tracking, consisting of a visible, an infrared, and a hyperspectral sensor. The system makes full use of its multisensor information collection abilities; furthermore, it can actively control different sensors to collect additional data, according to the needs of the real-time target recognition and tracking processes. This level of integration between hardware collection control and data processing is experimentally shown to effectively improve the accuracy and robustness of the target recognition and tracking system.

## 1. Introduction

With the rapid development of image processing and computer vision, visual object recognition and tracking techniques have been widely used in the military, aerospace, scientific exploration, astronomical observation, and video surveillance fields; over the years, it has also become a hot research topic in the fields of automatic control, computer vision, and pattern recognition. In most target recognition and tracking systems, data collection and processing are completely separated tasks; sensors are only used to collect data, and the data processing system is only used to process the collected sensor data. As a result of that separation, if data are found to be incomplete or ambiguous during processing and there are no available supplementary data to mitigate those deficiencies, the accuracy and robustness of the recognition and tracking processes may be insufficient for the desired applications. For example, to recognize and track a ship in a commercial harbor for traffic management of the harbor, the ship is a point-like target when it is far away, and furthermore, it may be blocked by other ship or be effected by complicated weather conditions. To overcome the incomplete or ambiguous data problem, some active multimodal sensor systems have been proposed. Cho et al. proposed a multisensor system for moving object detection and tracking in an urban driving environment. Radar, LIDAR, and vision sensors were used. The system could actively control all the sensors to collect or supplement target information and effectively detect and track target movement. The system obtained good performance results in actual driving conditions [1]. Tran et al. presented a target detection system with two PTZ (Pan Tilt Zoom) cameras. They used a simple image coordinate transformation to control the cameras. A wide-view camera was first used to perform target detection in real life scenes, potentially detecting several targets. The narrow-view camera was then cued (through the mentioned coordinate transformation) to actively collect high-resolution images of the targets whose resolution was lower than the defined threshold. The accuracy of the overall target detection system was thus much improved [2]. Cui et al. also proposed a binocular system using two PTZ cameras. A master camera tracked moving objects at low resolution and provided positional information to the slave camera. To solve the camera collaboration problem, planar geometric constraints were exploited; the slave camera was actively pointed towards the object at high resolution, and tracked it dynamically [3]. Considering that a PTZ camera tracking system can rotate and zoom in and out to keep the interesting object within its field of view, many investigations have focused on the study of active systems to overcome the drawbacks of static systems, which cannot acquire sufficient data of the regions of interest [4,5,6]. Qu et al. integrated a PTZ camera, a mirror, and a pan-tilt unit with a laser Doppler vibrometer (LDV), thus forming a multimodal sensing system to automate remote voice detection. Based on video analysis and triangulation of the target, LDV laser beam, and PTZ camera, the system could automatically select the best reflective surfaces, and actively point and focus the laser beam to the selected surfaces. This process can improve the LDV performance and efficiency in automatic remote hearing applications [7]. The above mentioned research works are good examples of how the combination of multimodal hardware data collection and software data processing can not only improve the accuracy of recognition and tracking systems, but also expand the target recognition and tracking range and increase system’s flexibility.

However, in the maritime surveillance field, hardware data collection and software data processing are two completely separate and independent parts [8,9,10,11,12,13,14]. Most of these literatures only use software data processing algorithms to improve the vessels’s detection rate and tracking rate and hardware sensor is just a data collection tool. For example, Szpak et al. used a background subtraction method and employ a real-time approximation of level-set-based curve evolution to demarcate the outline of moving vessels in the ocean [8]. Sullivan et al. detected vessels by an edge-enhanced spatiotemporal optimal trade-off maximum average correlation height filter [9]. Makantasis et al. designed a visual attention method that exploits low-level image features with an online adaptable neural network tracker, without making any assumptions about environmental or visual conditions [10]. The NATO Undersea Research Centre exploited an Automatic Identification System (AIS) for maritime surveillance using the distributed multi hypothesis tracking based on Kalman-filtering [11]. Makantasis et al. presented a vision-based system for maritime surveillance adopted an appearance-based approach to create visual attention maps that represent the probability of a target being present in the scene [12].Tran et al. applied dynamic fusion technique on background subtraction and saliency detection results for boat detection [13]. Teutsch et al. used support vector machines (SVMs) to distinguish between three object classes: clutter, irrelevant objects and suspicious boats, and obtain a rate of 97% correct classifications [14]. In summary, all of these literatures directly used the data collected form the sensor and applied mature algorithms such as SVMs to improve the target detection rate and tracking rate, and did not consider the interaction with the sensor to supplement the data and eliminate ambiguous judgments, to improve the detection rate and recognition rate.

In this paper, an active multimodal sensor system for target recognition and tracking is proposed and discussed. Different from passive multi-modal sensor system whose main task is collection data and transfer data to data processing software, the active multi-modal system is able to adjust sensors’ attitude to get supplementary information during data processing. Different modal sensors will be commanded to collect supplementary data from the target in order to eliminate ambiguous recognition and tracking judgments because of flawed data; this helps to confirm the results, improve the overall accuracy, and increase the robustness of the system.

The active multimodal sensor system consists of a visible sensor, an infrared sensor, and a hyperspectral sensor, working together to improve the target recognition and tracking accuracy. The visible image provides high contrast and rich details, but its performance degrades severely under dark conditions. The infrared image has strong anti-interference capabilities and is highly convenient for target recognition, but it has low resolution and low contrast. The hyperspectral data contains information concerning the target surface material, but only one slit image can be obtained at a time. The system proposed control actively the slit of the hyperspectral sensor to aiming the target, thus collecting more accurate and comprehensive information pertaining to the target, and improving the accuracy of target recognition and tracking.

This paper makes two main contributions. First, we propose an active multi-modal sensor recognition and tracking system, in which the hardware data collection and software data processing can interact with each other. During data collection, the data processing algorithm will cue and control the sensors to optimize the target data acquisition (controlling the hyperspectral sensor to aiming the target). During data processing, different modal sensors will be commanded to collect supplementary information from the target, according to the requirements of the data processing system; this helps to confirm the recognition and tracking results, improve the overall accuracy, and increase the robustness of the system. Second, we used a hyperspectral sensor and a hyperspectral-data based recognition algorithm for the target recognition and target tracking. The spectrum of the target depends only on its composition and is not sensitive to the environment. Hence, the accuracy of a recognition and tracking method based on the spectrum is higher than that of methods based on images. In particular, when the target is far away and shape information is not available (the target is like a point, only a few or a few tens of pixels in size), the hyperspectral data can still be used to recognize and track it. The system proposed in this paper can recognize and track targets in challenging conditions, e.g., when there are two targets with the same shape in the scene, when the tracked target has been lost, or even when the wrong target is being tracked. The system is able to detect these situations in real time and go back to tracking the correct target. The situations mentioned above can be probed in the supplementary material “Active Multimodal Sensor System for Target Recognition and Tracking.mp4”.To the best of our knowledge, this is the first time to propose an active multimodal sensor system consists of a visible sensor, an infrared sensor, and a hyperspectral sensor, working together to improve the maritime target recognition and tracking accuracy.

The rest of this paper is organized as follows: Section 2 introduces the hardware components of the active multimodal recognition and tracking system, the key system characteristics, and the overall system workflow. In Section 3, several methods for multisensor information fusion are presented and discussed. In Section 4, a process and the constituent methods for multimodal target recognition is proposed. In Section 5, a process and the constituent methods for multimodal target tracking is proposed. In Section 6, experimental comparisons and validations are presented. Section 7 concludes the paper.

## 2. Conceptual System Design

**Figure 1:**
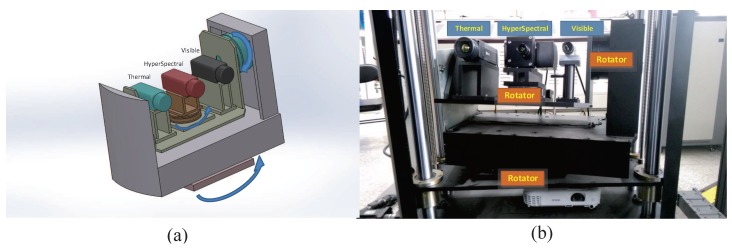
Multimodal sensor system: (**a**) System configuration; (**b**) Experimental setup.

Figure 1a shows a schematic view of the system. It consists of a visible sensor, an infrared sensor, a hyperspectral sensor, a rotator (located below the hyperspectral sensor), and a pan-tilt platform. The three vision sensors are fixed on the platform (visible light on the left, hyperspectral on the middle, and infrared on the right). A one-dimensional rotator is placed below the hyperspectral sensor, to allow it to perform two-dimensional scanning imaging. The sensors can be moved in two dimensions, with pan and tilt motions, so that the target can be locked in the image center of one of the sensors for accurate target recognition and tracking. A photograph of the experimental setup is shown in Figure 1b. The selected visible sensor is an acA1920-155uc camera produced by Basler (Ahrensburg, Germany), with a full frame resolution of 1920 × 1200, a full frame rate of 155 frames per second (fps), and a focal length of 16 mm. The used infrared sensor is an A615 camera produced by FLIR (Nashua, NH, USA), with a full frame resolution of 640 × 480, a full frame rate of 50 fps, and a focal length of 24.5 mm. The chosen hyperspectral sensor is a Hyperspec VNIR-N imaging sensor produced by HeadWall (Bolton, MA, USA), with a slit width of 25 μm and a working wavelength range of 380–1000 nm divided into 250 bands. The detector is a Falcon 285 camera, produced by Raptor Photonics (Milbrook, Northern Ireland), with a full frame resolution of 1004 × 1002, a full frame rate of 30 fps, and a focal length of 12 mm. In addition, in order to ensure the synchronization of the images from different sensors and consider high frame rate compatible with low frame rate, the update rate of three sensor is set to the same value of 30 fps and the software multithread synchronization mode is used to synchronous collect data. In the future, to make sure strictly synchronization collection, the hardware synchronization mode could be used.

**Figure 2:**
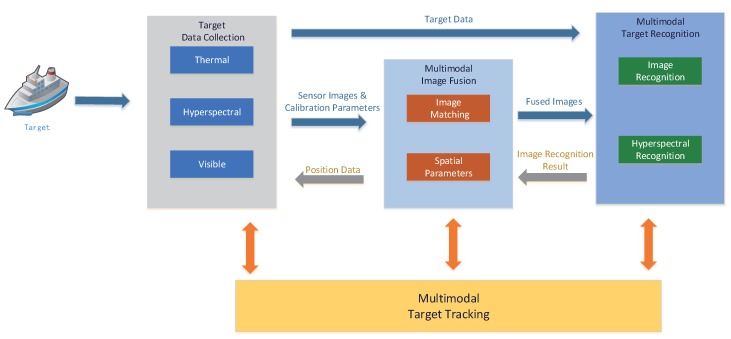
System workflow.

The proposed system workflow includes four main functional components (see Figure 2): target data collection, multimodal image fusion, multimodal target recognition, and multimodal target tracking. The target data collection module is used to collect visible, infrared, and hyperspectral data of the target, using the above three sensors. Multimodal optical images (visible and infrared images) are fused in the multimodal image fusion module, and at the same time the distance between the target and the system can be calculated by triangulation between the visible sensor, the infrared sensor, and the target. The multimodal target recognition module is used to identify the existing target; the module performs target recognition based on the optical images (visible, infrared, and visible-infrared-fused) and hyperspectral data. Because of the hyperspectral sensor’s imaging mode (only one slit image can be obtained at a time), it is necessary to drive the rotator below the sensor to make the imaging beam point at the target; therefore, the spatial parameters obtained by the multimodal image fusion module are needed here. Having done that, the system can collect accurate hyperspectral data of the target and perform a further confirmation by hyperspectral recognition. The multimodal target tracking module is used to track the detected target. This module cooperates with the data collection, fusion, and recognition modules. In particular, the data collection module will be called to actively collect visible or infrared images and target hyperspectral data whenever the target tracking module needs supplementary data. With this supplementary data, the system can obtain further confirmation on the target’s nature (true or false), and ensure tracking accuracy and robustness. When the target is lost, the multimodal recognition module will also be called, to re-recognize the true tracking target in full view, thus helping the tracking module recover the true target track. The above-described overall workflow ensures the robustness and anti-interference resilience of the system tracking capabilities.

From the above description of the recognition and tracking system workflow, we can conclude that the active multimodal recognition and tracking system proposed in this paper is a closed loop system, which combines and merges together the hardware collection control and software data processing. The key characteristics of this system are: fast matching of the visible and infrared images, and a resulting fast cueing of the hyperspectral sensor’s imaging beam (extended line of the lens optical axis) from the target; active target recognition with collaborative multimodal sensors; finally, active target tracking with collaborative multimodal sensor control and data collection. Once the visible sensor is completely disabled, we will not be able to get information on the target distance, and the system will not be able to operate in active mode; at this point, the infrared sensor will still operate and the system will go into the monitoring mode, which is further explained later, when describing the multimodal tracking process.

## 3. Multimodal Image Fusion

Fusing the data collected by the three sensors is a challenging task, because the data collected by the visible and infrared sensors are quite different in the quantity and position of feature points both on the target and in the background. In addition, the fact that only one slit image can be obtained from the hyperspectral sensor at any given time is also a challenge. In this paper, a priori information on the fixed relative position among the three sensors is used. The intrinsic parameters of all three sensors and the extrinsic parameters of the sensor set are first calibrated by both monocular and stereo camera calibration procedures. The visible and infrared images are then rectified using the stereo calibration results, so that the pixel rows of the visible and infrared cameras are exactly aligned with each other before the image matching process. Having done that, we can search the relative feature points only on the epipolar lines, and thus accelerate the multimodal matching process, finally completing the multimodal image fusion.

### 3.1. Camera Calibration

The calibration flow for the three sensors consists of two steps. The first step is to collect target images with all three sensors. Jean-Yves Bouguet’s method is then used for monocular camera calibration [15], and the intrinsic parameters of each sensor are calculated. The results of the calibration performed on the experimental system are shown in Table 1. Extrinsic calibration is carried out one and the results is shown in Table 2, are in full compliance with the physical installation parameters of the three sensors.

### 3.2. Image Fusion of the Visible and Infrared Images

**Figure 3:**
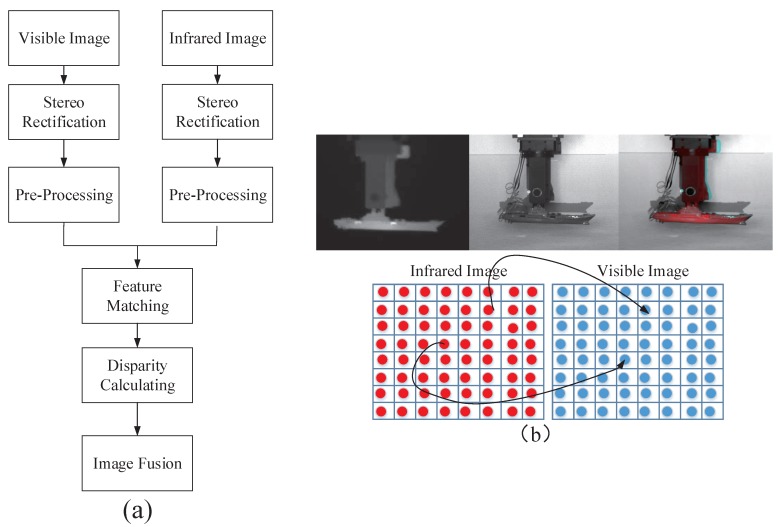
Fusion of the visible and infrared images: (**a**) Workflow; (**b**) Fused image.

The workflow of the fusion process for the visible and infrared image pairs is shown in Figure 3a. The 3D reconstruction algorithm [16] of binocular stereo vision modal is adopted in this paper. As a first step, the infrared image’s resolution is extended to be same as visible image by pixel interpolation method. Second, both images undergo stereo rectification; after extracting keypoints from a visible rectified image, then searching the corresponding points on the epipolar lines from a rectified infrared image, the disparity of the two images can then be calculated according to these matched keypoints pairs. Considering that the color red is related with heat, the image fusion is to make target object more obvious to detect and tracking. The fused image is then obtained by replacing the red channel intensity of the visible image with the gray value of the corresponding point of the infrared image (see Figure 3b). The distance to the target is calculated by triangulation between the visible sensor, infrared sensor, and target, based on the essential disparity of the visible and infrared image pair.

### 3.3. Image Fusion of the Hyperspectral and Infrared Images

**Figure 4:**
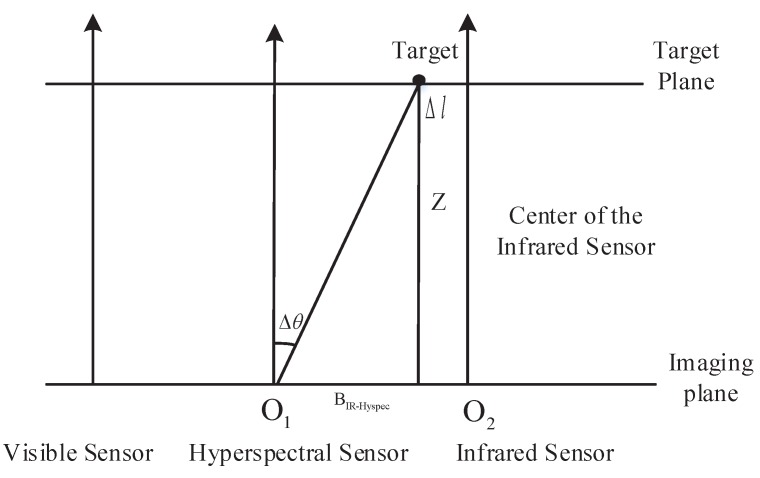
Fusion of the hyperspectral sensor image.

Using the extrinsic parameters (R and T) for the three sensors presented in Section 3.1 and the disparity between the visible and infrared image pairs calculated as discussed in Section 3.2, the distance to the target can be obtained by [17]

(1) $$z=\frac{f_{vis}\times B_{vis-IR}}{\Delta x}$$

where $f_{vis}$ is the focal length of the visible sensor (calculated in Section 3.1), Δx is the disparity between the visible and infrared images calculated by the image matching process, and $B_{vis-IR}$ is the baseline distance between the visible and infrared sensors. The values of $f_{vis}$ and $B_{vis-IR}$ were presented in Subsection III-A. The distance *z* between target and system can thus be calculated using Equation (1). Taking into account that the visible sensor may be affected by weather effects such as fog and lead to lower accuracy, the hyperspectral sensor will be mapped to the IR sensor. Based on the geometry relation between the hyperspectral sensor, target, and infrared sensor (see Figure 4), the rotation angle Δθ of the hyperspectral sensor can be calculated as follows:

(2) $$\Delta \theta =a\tan(\frac{O_{1}O_{2}-\Delta l}{z})$$

In (2), $O_{1}$$O_{2}$ is the baseline distance $B_{hyper-IR}$, *a* is the coefficient of pangle conversion into radian, *z* is the distance between target and system calculated by (1), and Δl is the horizontal distance between the optical center of the infrared sensor and the target, which can be calculated by

(3) $$\Delta l=\frac{d\cdot z}{f_{inf}}$$

Here *d* is the pixel distance between the target and the image center of the infrared sensor, *z* is calculated by (1), and $f_{inf}$ is the focal length of the infrared sensor, presented in Section 3.1. The system will then use the rotator installed below the hyperspectral sensor to perform a rotation of Δθ, thus aligning the imaging slit of the hyperspectral sensor with the target; the target’s hyperspectral data collection can then start. In addition, the center of the infrared sensor will be locked on the target when tracking. To ensure that the correct target data is being collected, the hyperspectral and infrared sensors should be bound by imposing that Δl=0. Finally, the hyperspectral and infrared images are fused while tracking.

## 4. Multimodal Target Recognition

**Figure 5:**
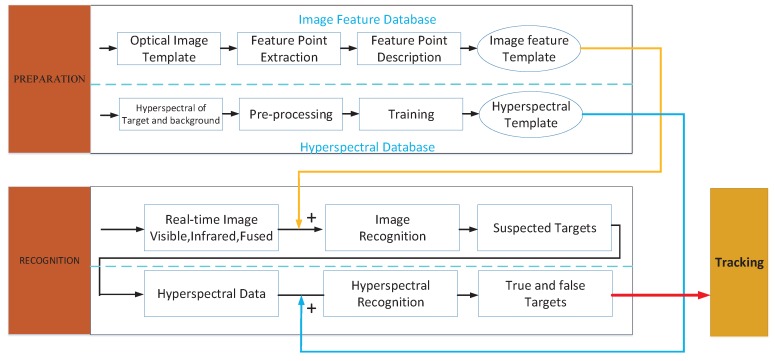
Multimodal target recognition.

The visible image, infrared image, visible-infrared fused image, and hyperspectral data all contain information concerning the reflectance distribution, emissivity distribution, and target surface material. All the above information can be used in the multimodal recognition process. In this paper, after image feature and hyperspectral database establish, the recognition based on optical data is performed firstly. Considering low success ratio of recognition based on optical data, we must use further information of the target. In order to collected target’s hyperspectral data, the hyperspectral sensor will be rotated Δθ to point at the targets, the hyperspectral data from target will be processed to choose the true target-the one that is most similar to the modal target in the hyperspectral database-and flag it. With the above process, the multimodal sensor system actively controls the sensors to collect reflectance, emissivity, and surface material related information, and identify the most accurate target step by step, with improved target recognition accuracy. The overall block diagram of the multimodal target recognition phase is shown in Figure 5. The workflow includes the following two steps: establishment of a target model database, and real-time target recognition.

*Step1*: Before target recognition, the template target databases must be created. In this study, both a feature database (containing visible, infrared, and fused image derived information) and a hyperspectral database are used. The feature database creation process is shown in Figure 5. First, quantities of visible and infrared target image pairs are collected, and the methods discussed in Section 3 are used to obtain fused images and supplementary data. After that, feature point extraction and description is carried out for those three kinds of images. Finally, the feature point descriptors of each target are saved to the image feature database. The hyperspectral database creation process is also shown in Figure 5. A large number of hyperspectral data of the target and background are first collected, and representative data of both are extracted, to be used as standard hyperspectral data. Filtering and denoising are executed next. The obtained data are labeled according to which class they belong to: target or background. A support vector machine (SVM) algorithm is then used and trained with the standard hyperspectral data, and the obtained results are fed into the hyperspectral database [18]. The SVM algorithm is supervised learning model with associated learning algorithms that analyze data used for classification and regression analysis. Given a set of training data, each marked as belonging to one or the other of two categories, an SVM training algorithm builds a model that assigns new examples to one category or the other, making it a non-probabilistic binary linear classifier.
*Step2*: The workflow of the real-time multimodal target recognition step includes two recognition phases, as shown in Figure 5: a pre-recognition phase, based on optical imaging, and a further recognition phase, based on hyperspectral data. The optical data is utilized to do preliminary recognition, but the recognition based on optical data relies on the target appearance. In the case of similar target interference, the recognition success rate is rather low, so we take advantage of the hyperspectral data to do further recognition that would increase recognition success rate. These will be further described below.

### 4.1. Optical-Image Based Recognition

**Figure 6:**
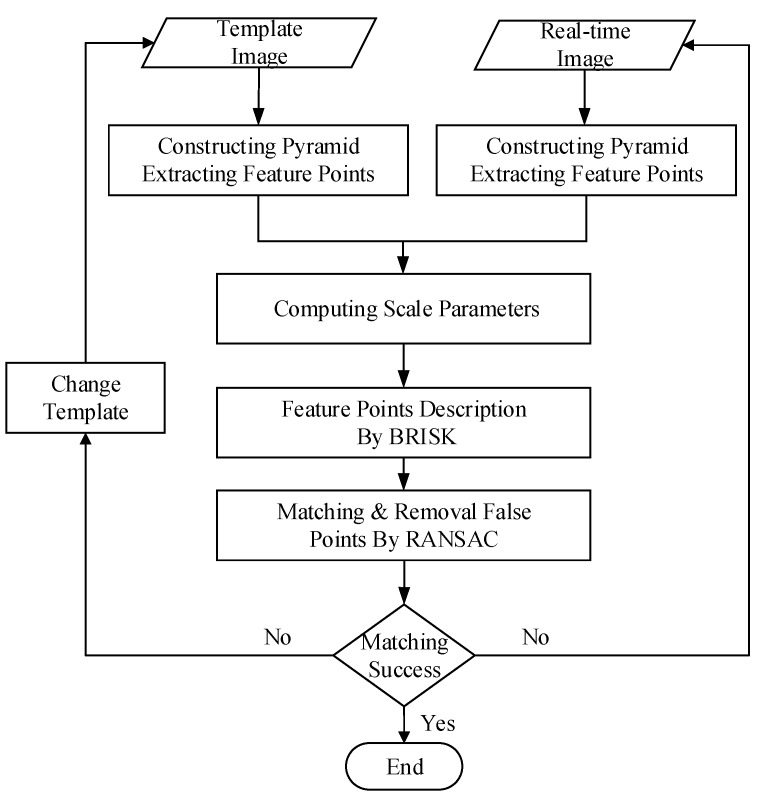
Optical-image based target recognition.

To ensure the image target recognition accuracy and decrease the recognition delay, a feature detection algorithm with adaptive scale selection is presented in this paper, which uses information content quantization in the scale space representation. The algorithm is based on scale-invariant feature points matching method which generally includes three steps: feature extraction, feature description, and feature matching [19]. The feature detection algorithm in this paper is shown in Figure 6. The full detection process consists of five steps:

1. The template image and the real-time image are processed to construct a pyramid scale space with same methodwhich use the features from the accelerated segment test (FAST) algorithm to extract feature points [20]. The number of FAST feature points is adopted as the image information contents in scale space. For each location on the circle with a radius of *r* pixels, the pixel *q* at this position relative to *p*, denoted by p→q, can be classified to one of the three states: darker, similar and brighter.
  (4) $$S_{p\to q}=\left\{\begin{array}{cc} d,\;I\left(q\right)\leq I\left(p\right)-\epsilon_{d} & \left(\mathrm{darker}\right)\\ s,\;I\left(p\right)-\epsilon_{d}\leq I\left(q\right)\leq I\left(p\right)+\epsilon_{d} & \left(\mathrm{similar}\right)\\ b,\;I\left(p\right)+\epsilon_{d}\leq I\left(q\right) & \left(\mathrm{brighter}\right) \end{array}\right.$$
  For any discrete image *I*, I(q) is the gray value of each pixel on the circle and I(p) is the gray value of candidate point *p*, $\epsilon_{d}$ is a gray threshold. The FAST detector classifies the candidate point *p* as a corner if there exists a set of *N* contiguous pixels in the circle which are all brighter than the intensity of the candidate pixel I(p) plus a threshold $\epsilon_{d}$, or all darker than $I\left(p\right)-\epsilon_{d}$. *N* is set to larger than a setting threshold *n* (*n* is chosen to be 12).
  Let *P* be the set of FAST keypoints extracted from the discrete image *I* :
  (5) P={p∈P∣N≥n}
  Then, the information content of the discrete image *I* can be defined as $E=\left|P\right|$, where $\left|P\right|$ indicates the number of elements in the set *P*.
2. The appropriate pyramid scale parameters are determined by the differences in information contents. The information content of L(x,y,σ(o,s)) is descripted by $inf_{s}$, then the difference between two adjacent Gaussian images can be expressed as $diff\_inf_{s}$.
  (6) $$diff\_inf_{s}=inf_{s}-inf_{s+1}\;s\in \left[0,S-2\right]$$
  where the SIFT scheme is referenced to build a pyramid including *O* octaves, and each octave is divided into *S* intervals. Each Gaussian image in the scale space of an input image I(x,y) is defined as L(x,y,σ(o,s)); σ is the absolute scale parameter.
3. The binary robust invariant scalable keypoints (BRISK) algorithm is used to obtain descriptors of the feature points extracted in the previous step [21].
4. Feature point matching is performed on the constructed scale-adaptive pyramid, and the RANdom SAmple Consensus (RANSAC) algorithm proposed by Fischler and Bolles is used for removal of false matching points [22].
5. The pyramid level is controlled by thresholding the number of matching pairs (i.e., stop matching and constructing when the number of matching pairs reaches the threshold). For a given initial grayscale image *I*, the Gaussian image in the pyramid is expressed as L(x,y,σ(o,s)) with o∈[0,O-1],s∈[0,S-1]. The initial image is L(x,y,σ(0,0))=I. For the remaining octaves, the Gaussian image at the first level of each octave L(x,y,σ(o,0)) must be down-sampled by a factor of 2 from the Gaussian image L(x,y,σ(o-1,S-1)) in the previous octave. The number of octaves is determined by the minimum number of pixels in the top-level smoothed image in the pyramid. For an initial grayscale image *I* with a size of width×height pixels, the minimum number at the top level is $2^{n}$, $n\in [0,\log_{2}\min\left(width,height\right)-1]$. The number of octaves *O* can be computed by Equation (7):
  (7) $$O=\log_{2}\min\left(width,height\right)-n$$
  If the target does not match the current template, a new template in the database is used to match the target, and the above five steps are repeated. Unlike conventional algorithms that match the feature points on a complete pyramid, here a simultaneous, joint construction and matching strategy is used: the pyramid construction will end as soon as an adequate number of correct matching pairs has been obtained. Not only does this allow for lower computational costs, but also the problem of the images becoming increasingly rough from bottom to top-which results from the quantitative processing of the pyramid-can be avoided.

In principle, our method not only extracts feature points with lower time cost, but also maintain scale invariance. BRISK descriptor could further lower time cost, and also maintains the rotation invariance properties. In summary, our method is more suitable for real-time optical recognition of sea target.

### 4.2. Hyperspectral-Data Based Recognition

To refine the pre-recognition obtained by optical imaging, the suspected targets undergo a second recognition phase (hyperspectral-data based), as previously discussed. A supervised classification method is adopted for recognition of the obtained hyperspectral data. To get the templates, first we artificially select the region of interest on the pre-shot hyperspectral image; then we extract the data from the region and divide them into target and non-target data; finally, we process them using an SVM machine-learning method to generate the hyperspectral templates. In practical application, hyperspectral sensor’s imaging beam is pointed at target to get spectral data automatically. According to the hyperspectral templates and real-time targets’ spectral information, the SVM is also used to recognize and classify suspected targets which is based on hyperspectral-data. Specifically, 14,870 hyperspectral data points are used here to train the SVM. The hyperspectral data is then filtered and denoised.

## 5. Multimodal Target Tracking

**Figure 7:**
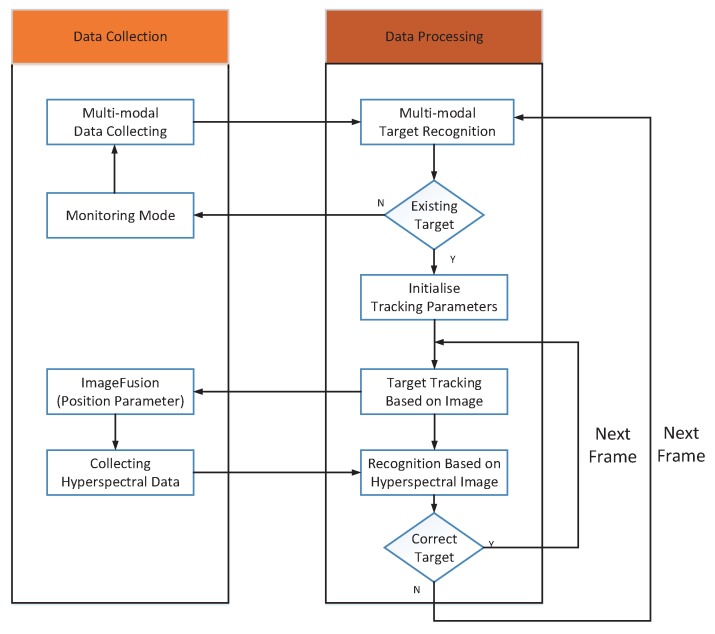
Multimodal target tracking.

In this paper, the multimodal information collected by the visible, infrared, and hyperspectral sensors is comprehensively utilized. Multimodal information is collected and actively supplemented during tracking. Optical image recognition and hyperspectral confirmation are included into the tracking process, to ensure that the true target is being tracked. Our approach can also find and recover the true target when the target is lost or when it is found that a false target is being tracked, by using the multimodal recognition module. The multimodal target tracking method proposed in this paper can resist a variety of interference sources and achieve long-time continuous tracking. The multimodal target tracking workflow is shown in Figure 7.

The multimodal target tracking includes two modules: data collection and data processing. Data collection and data processing work together to track target. Multimodal information could be collected and actively supplemented during target tracking. First, the multi-modal system starts with collecting data, and then the multi-modal target recognition block identifies the target and target’s position in image. After a target judgement, if there is target existing in field of the view, the image with the initial recognition box will be sent to the tracking procedure, and the target will be locked to the center of the infrared sensor. If not, the system will enter a monitoring mode. In this mode, the system will detect targets coming into view at any moment. If the target is detected, the multimodal recognition and tracking processes will be restarted. Once the tracking procedure starts executing, the parameters of tracking parameters will be initialized by the data from recognition box, and next frame tracking will be initiated with these parameters. After tracking parameters initialization, a tracking algorithm that presents a framework for adaptive visual object tracking, based on the structured output prediction (Struck) proposed by Hare et al., is used here for target tracking [23], and the tracking starts proceeding. Concurrently, the system will get position parameter from fusion image of the visible sensor and inferred sensor and control the hyperspectral sensor to collect the target’s hyperspectral data. The recognition based on hyperspectral data is used to confirm that the tracked target is the true one. If the target is indeed the true one, the next frame will be sent to the tracking module for processing. If not, the multimodal target recognition module will be restarted to detect and recognize suspected targets in the whole image again, and the so-obtained suspected targets will be sent to the hyperspectral recognition module. Once the new is classified as true target, its position will be sent to the tracking module again, which will then reinitialize tracking parameters and restart tracking. If not, the process will repeat. In this way, data collection can be control at any time to capture supplementary image or hyperspectral data for target tracking task, which increase the accuracy and robustness of the tracking processes.

It is worth noting, the Struck tracking algorithm need set tracker searching radius artificially, the parameter is lower, the time cost is higher, but the accuracy of algorithm is higher, a suitable value could be set according to application requirement and a few of tests.

## 6. Experiments

To test the reliability and accuracy of the proposed recognition and tracking system, an experimental platform was built. The sensor description and arrangement has already been introduced in Section 2.

In this study, we conducted three different types of experiments: laboratory simulations, comparisons between algorithms, and realistic experiments.

For the simulation experiments, the experimental targets were two scaled-down ship models. The size of both models was 315 × 78 × 65 mm${}^{3}$. A heating device was used to heat the models from 30 to 150 ${}^{\circ }$C. In addition, different surface materials were used for the two models. The experimental system could control the movement of the experimental targets, with both three-dimensional translational motions and two-dimensional rotational motions. Active target recognition and tracking was performed while the targets were moving.

We also performed comparisons between selected state-of-the-art algorithms and our algorithm, using simulated data, a standard dataset, and realistic data. The standard dataset (Crowds, Panda, and Sylvester) used to test tracking algorithm in this paper has included the ground truth data, so we could compare the ground truth data with tracking trajectory of the proposed method to evaluate tracking algorithm directly. The ground truth of the realistic data is marked artificially by ourselves, and then we compare the ground truth data marked artificially with tracking trajectory of the proposed method to evaluate the algorithm.

Finally, a series of realistic experiments were conducted in the port. The targets were the real ships sailing in the port, the distance between the target and the system was between 0.5 and 10 miles, and the experiments were conducted from 0.5 h before sunrise until 1 h after sunset.

Using the above described experimental setup, we performed a number of experiments, recognizing and tracking different numbers of targets in different states, using the single sensor mode, the multimodal sensor passive mode (the sensors were fixed, data collection and data processing are separated tasks), and the multimodal sensor active mode(data collection and data processing are cooperated) proposed in this paper, to verify the accuracy of recognition and tracking. The system software was implemented in VS2010, which runs on an Intel(R) Core(TM) 3.4 GHz CPU with 4 GB of RAM.

### 6.1. Target Recognition Algorithm Comparison

The standard Mikolajczyk dataset [24] was used to evaluate our method and other algorithms in terms of four types of distortions, namely changes in the viewpoint, different light conditions, blur, and JPEG compression. Five image pairs with increasing amount of distortion were used. The parameters in the detectors were the same as in the experiments above.

**Figure 8:**
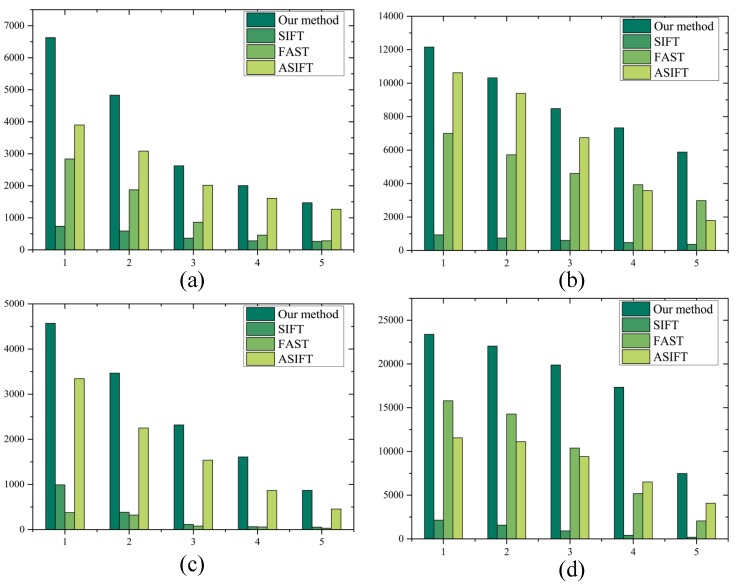
Number of correct matches achieved by our method, SIFT, FAST, and ASIFT under four types of distortions in the standard Mikolajczyk database. (**a**) Blur. (**b**) Light. (**c**) Viewpoint. (**d**) JPEG compression.

As shown in Figure 8, all the four algorithms proved to be robust against blur, with our adaptive multiscale feature detection algorithm and ASIFT [25] producing more effective matches than SIFT [26] and FAST [20]. Under different conditions of light, all the algorithms tend to be robust as well. When the viewpoint changes, both SIFT and FAST have limited robustness while our method and ASIFT showed high performance. SIFT also has difficulty in dealing with changes in compression, while the other three methods are very robust and found sufficient correct matches. ASIFT performed well through all the database tests, even though our method exhibited greater robustness and yielded more valid matches with limited numbers and scale-uniform feature points.

To summarize, experiments were conducted to show that our method can simultaneously adapt to changes in location, scale, illumination, and viewpoint, and can generate large numbers of features that densely cover the images over a large range of these variations. Our approach significantly improves the results in the presence of environmental changes and can be thus very useful for various applications.

### 6.2. Target Recognition Experiments

To test the accuracy of the active multimodal recognition, first, we placed the two ship models in the system’s field of view. The models were precisely identical, but there were slight differences in surface color (not distinguishable with the visible sensor). Several temperature combinations for the pair of models were tested, including the following temperature pairs: [25 ${}^{\circ }$C (unheated), 25 ${}^{\circ }$C], [25 ${}^{\circ }$C, 75 ${}^{\circ }$C], [75 ${}^{\circ }$C, 25 ${}^{\circ }$C], [75 ${}^{\circ }$C, 75 ${}^{\circ }$C], [25 ${}^{\circ }$C, 150 ${}^{\circ }$C], [150 ${}^{\circ }$C, 25 ${}^{\circ }$C], [75 ${}^{\circ }$C, 150 ${}^{\circ }$C], [150 ${}^{\circ }$C, 75 ${}^{\circ }$C], [150 ${}^{\circ }$C, 150 ${}^{\circ }$C]. We specified a priori one of the ship models as the true target. The image feature database and hyperspectral database were created, based on different angles and different distance views of the two targets. Having completed that, the recognition performance under different temperature conditions was evaluated in single sensor mode (for both the visible and infrared sensors), multimodal sensor passive mode, and multimodal sensor active mode (the mode proposed in this paper).

Then, in order to investigate the application of the multimodal system, we conducted a series of experiments in the port. The process was exactly the same as before: Prior to the field experiment, feature and hyperspectral databases of cargo ships were created, based on different angles and distance views of the targets. Then, the recognition performance was evaluated in single sensor, multimodal sensor passive, and multimodal sensor mode at different times throughout a day.

**Figure 9:**
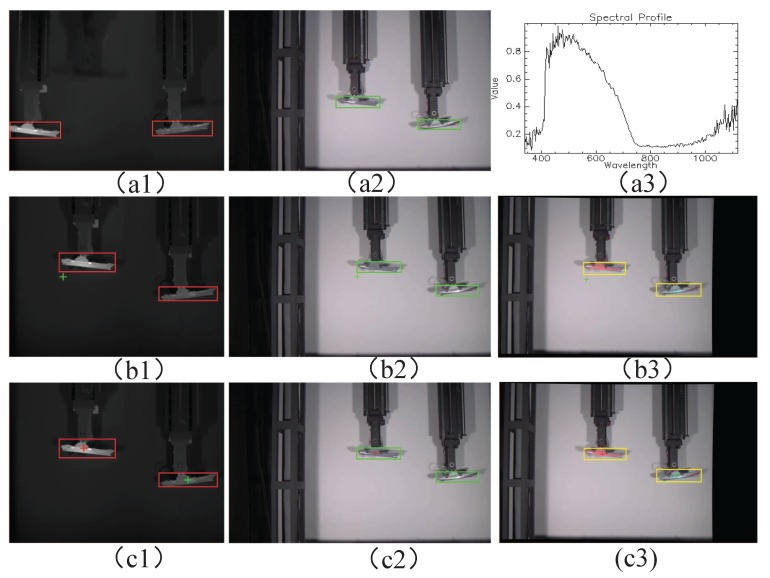
Indoor recognition results: Labels 1 to 3 correspond to the infrared, visible, and fused images, respectively. (**a1**–**a3**) Single sensor mode; (**b1**–**b3**) Multimodal sensor passive mode; (**c2**–**c3**) Multimodal sensor active mode.

**Figure 10:**
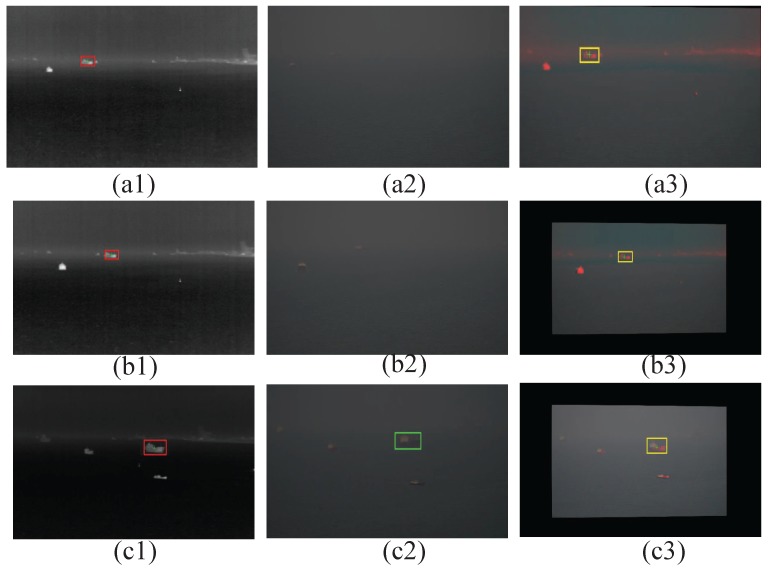
Outdoor recognition results: Labels 1 to 3 correspond to the infrared, visible, and fused images, respectively. (**a**–**c**) indicates a gradual increase in visibility.

The obtained results are shown in Figure 9 and Figure 10. In these figures, a red cross near the ship model identifies it as the true target, whereas a green cross positively identifies it as a false target. From this figure, we can see that targets could be detected using single sensor mode or multimodal sensor passive mode, but the true target could not be recognized because two target have same shape and color. On the other hand, the multimodal sensor active mode was capable of not only detecting the targets, but also identifying the true target because the active mode can control the slit of the hyperspectral sensor to aiming the target and capture the hyperspectrum data of the target for recognition.

We also made a comparative analysis of the target recognition accuracy for the three modes and different algorithms (SIFT, FAST, and ASIFT), to obtain an objective quantitative evaluation. Target recognition has many different evaluation indexes [27,28]; in this paper, the ratio of successful recognition results to the total test instances was considered, and is defined here as the recognition rate (RR)

(8) $$RR=\frac{S_{s}}{A}\times 100\%$$

where *A* is the total number of experiments and $S_{s}$ is the number of successful real-time recognitions (when the overlapping area of the recognition box and the ground truth is greater than 80%). Erroneous classification of false targets as being the true target is palways considered a recognition error, no matter how much overlapping area the recognition box exhibits.

Table 3 shows the recognition rate results for the three recognition modes. As can be seen, single sensor recognition is affected by the targets in the scene, and has a very low recognition rate. The multimodal sensor passive mode (using the above four algorithms and calculating the average) exhibits a low recognition performance, because the data collected by the hyperspectral sensor is not always reliable. Using the multimodal sensor active mode (with our proposed algorithm), however, allows for a proper and effective collection of the target’s reflectance distribution, emissivity distribution, and surface material information, and a very high recognition rate can therefore be achieved.

### 6.3. Target Tracking Algorithm Comparison

**Figure 11:**
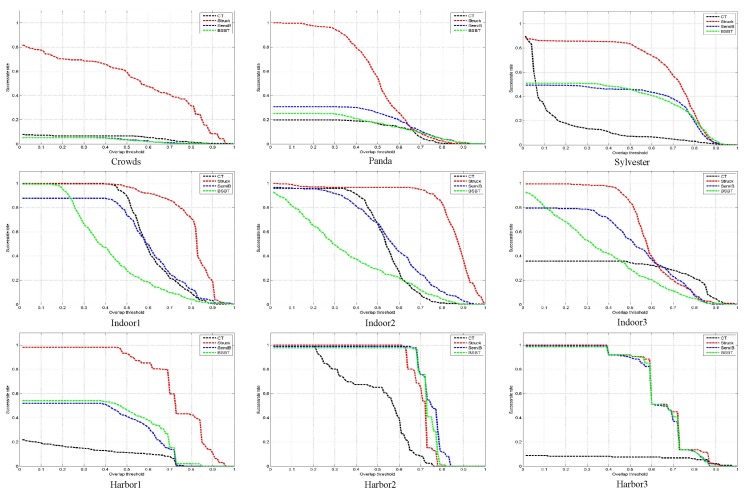
Tracking success rates for different algorithms (Red line-Struck, green-BSBT, black-CT, blue-SemiT).

**Figure 12:**
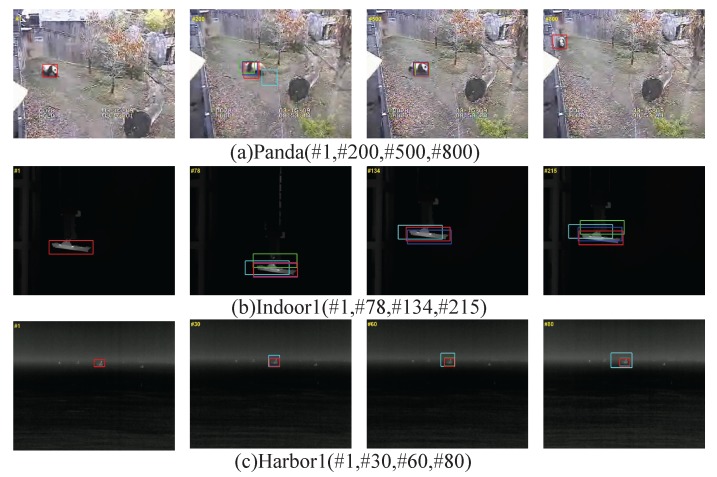
Screenshots of some tracking results.

In order to verify the effectiveness of the algorithm, we selected three representative sequences of images for the standard dataset (Crowds, Panda, and Sylvester), the indoor simulation (Indoor1, Indoor2, and Indoor3) and the outdoor sea experiment (Harbor1, Harbor2, and Harbor3). In addition, the algorithm used in this paper is compared with three popular algorithms, namely the CT [29], BSBT [30], and SemiT [31] algorithms. We used the parameters recommended by the original algorithm to carry out the index analysis, and got the average performance as the final performance index. In terms of algorithm comparison, we followed the standard presented in Section 6.2. The comparison results are shown in Table 4 and Table 5 and Figure 11 and Figure 12. Table 4 lists the average position error of each algorithm involved in comparing different image sequences.

To quantify the performance of the tracking algorithm, the tracking success rate [32] is used as an objective tracking accuracy evaluation index. The underlying success measure is

(9) $$S=\frac{∥r_{t}⋂r_{a}∥}{∥r_{t}⋃r_{a}∥}\times 100\%$$

where $r_{t}$ is the real-time tracking box, $r_{a}$ is the ground truth, and the ⋂ and ⋃ operators represent the intersection and union of the two boxes; the operator represents the number of elements in the corresponding region. The tracking algorithm performance is then measured by calculating the percentage of frames with overlapping rates *S* over a given threshold $t_{0}$, in a tracking image sequence. The higher the index, the better the performance of the tracking algorithm ($t_{0}$ is taken as 50% in this paper).

As can be seen, the algorithm we used (Struck) had a good performance. Since the average center position error only reflects the difference in the Euclidean distance between the real frame and the center of the tracking frame, it is not very effective to measure the performance of the tracking algorithm when dealing with targets of different scales. The tracking success rate is an analysis of the overlap ratio between the two frames, which can reflect the adaptability of the tracking algorithm to the change of scale due to target movement, and the tracking time also has been added to Table 4. Table 5, shows the tracking success rate of the algorithm when the overlap ratio is more than 50% of the given threshold. It can be seen from the analysis of the table that the algorithm used in this paper produced better results than the others for the nine sequences of pictures involved in the test. The success rate curves for the four algorithms and screenshots of some tracking results are given in Figure 11 and Figure 12. It can be seen from the figures that the performance of the algorithm we used is far superior to that of the other algorithms.

### 6.4. Multimodal Target Tracking Experiments

To test the accuracy and performance of the active multimodal tracking mode, we placed the two ship models in the system’s field of view. As mentioned above, the models were precisely identical, but there were slight differences in surface color (not distinguishable with the visible sensor). Both models were heated to 150 ${}^{\circ }$C. We specified one of the ship models as the true target a priori. The image feature and hyperspectral databases were created, with several different distances and view angles. Several different types of motions were used for the models, including linear uniform and sinusoidal types of motion, and the three previously discussed sensor modes (single sensor mode, multimodal sensor passive mode, and multimodal sensor active mode, all using the Struck algorithm) were used to verify tracking performance.

**Figure 13:**
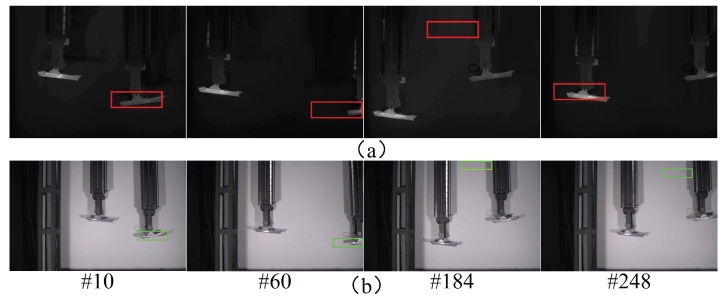
Tracking results for the single sensor mode (#xx designates the frame number). (**a**) Infrared images; (**b**) Visible images.

**Figure 14:**
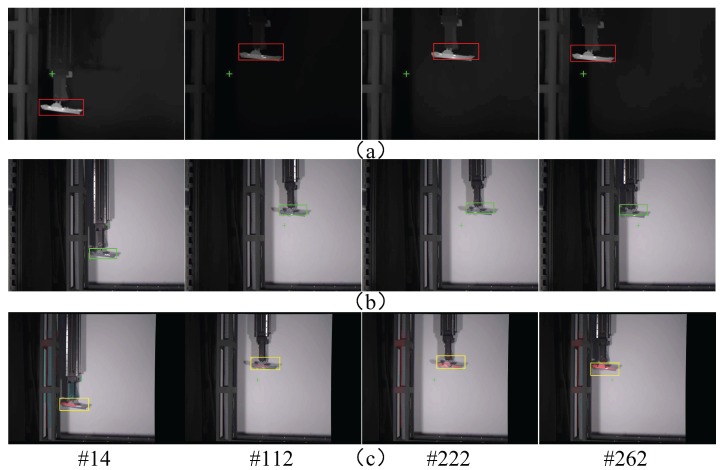
Tracking results for the multimodal sensor passive mode; the hyperspectral sensor is horizontally aligned with the infrared sensor, and with no rotation (#xx designates the frame number). (**a**) Infrared + hyperspectral; (**b**) Visible + hyperspectral; (**c**) Fused + hyperspectral.

**Figure 15:**
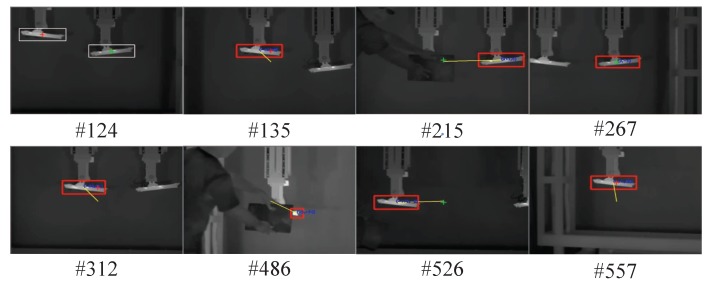
Tracking results for the multimodal sensor active mode (#xx designates the frame number). (The infrared image center-starting point of yellow line-is centered on the target).

The results obtained for the three sensor modes are shown in Figure 13, Figure 14 and Figure 15. As can be seen in Figure 13, when the target was tracked in single sensor mode, the initially acquired target was likely to be the wrong one, which resulted in the continuous tracking of the false target. In addition, when the tracked target was lost or blocked, the sensor could not re acquire the true target. A slightly different situation can be observed in Figure 14, for the multimodal sensor passive mode; in this case, the initial tracking accuracy was slightly improved, and the possibility of directly tracking the false target was reduced. When the target was lost or blocked, the system could occasionally find the real target to continue tracking, by using the three available images (infrared, visible, and fused). However, target re-acquisition was fortuitous and not reliable, because the target surface material information collected by hyperspectral imaging was not being effectively utilized. Finally, when tracking was performed in the multimodal sensor active mode, the system could reliably find and track the true target and recover from target losps or blocking situations. This can be seen in Figure 15; the red cross is used again to identify the true target in this figure, whereas the green cross represents a positive identification of a false target. A tracking success rate is also defined when the system does not track the target because only the false target is visible.

Table 6 shows the tracking success rates for the three kinds of operation modes. We can see that the single sensor mode has very low success rates. Some improvement in the success rate can be obtained by using the multimodal sensor passive mode, but this is not a stable configuration; only when the slit of the hyperspectral sensor is pointing at the target is the tracking success rate higher. However, very high success rates-which are always high and stable-can be obtained by using the multimodal sensor active mode to track the target because the slit of the hyperspectral sensor is control to pointing at the target always. In addition, from the large number of performed experiments we verified that the success rate of the single sensor and multisensor passive mode will considerably decrease when dealing with long tracking times (and decrease further as the tracking time increases), and that the system is prone to lose the target in those conditions. In active mode, on the other hand, very high success rates can also be achieved in those situations.

## 7. Conclusions

In this paper, we proposed an active multimodal sensor recognition and tracking system, consisting of a visible sensor, an infrared sensor, and a hyperspectral sensor working together to improve the target recognition and tracking accuracy. First, the system recognizes and tracks targets not only by passively combining the data collected by the three imaging sensors, but also by actively cueing and controlling the sensors to optimize target data collection. Second, a hyperspectral-data based recognition algorithm is used in the target recognition and target tracking process. The proposed approach solves the problems of external interference susceptibility and environmental dependence existing in single sensor or in general passive multimodal recognition and tracking systems. The data collected by the visible, infrared, and hyperspectral sensors are first fused using stereo calibration and FAST feature matching. Having done that, the multisensor information is fully utilized, and the sensors are controlled to actively collect additional, supplementary target information. The target recognition and tracking algorithms are carefully combined with the sensor data collection, so that the sensors can be accurately controlled to collect additional target data during the recognition or tracking phases, as often as needed, to further ensure accuracy and robustness. This type of solution, combining the hardware collection control and the data processing is shown to be capable of effectively improving the accuracy of target recognition and tracking systems, and also improve the anti-interference capabilities of these systems; as such, this approach may open a new technical path for target recognition and tracking systems development.

## Figures and Tables

**Table 1:** Intrinsic parameters.

Parameters		Visible Sensor	Infrared Sensor	Hyperspectral Sensor
Focal Length (pixel)	X	2800.8796	1451.5284	1556.703
	Y	2791.2893	1451.4501	1508.542
Principal point(pixel)	X	884.0507	312.1185	292.368
	Y	640.0548	253.799	198.586
Radial distortion	first-order	–0.12848	–0.09549	–0.63794
	second-order	0.08027	1.44284	1.59121
Tangential distortion	first-order	0.00062	0.00127	–0.00242
	second-order	–0.01071	–0.00171	0.00694

**Table 2:** Extrinsic parameters.

Parameters	Visible & Infrared			Infrared & Hyperspectral		
Rotation Matrix(mm)	0.99984	–0.01252	0.01256	0.99981	0.00591	0.01807
	0.01253	0.99992	0.00061	–0.00601	0.99996	0.00616
Translation Vector(mm)	0.01255	–0.00077	0.99992	–0.01804	–0.00627	0.99981
	–321.4846	–1.0623	5.0289	–188.04000	–0.42570	3.05072

**Table 3:** Recognition rate.

Source	Single Sensor (Average)				MSP	MSA
	Proposed	SIFT	FAST	ASIFT		
STT	91.3%	87.1%	52.3%	74.8%	93.4%	95.1%
STF	11.8%	9.1%	4.5%	5%	20.5%	95.2%
MT	51.1%	46.4%	32.7%	30.5%	55.6%	93.8%
Average	51.4%	47.5%	29.8%	36.8%	56.5%	94.7%
Average time cost(ms)	288	419	227	2987	—	—

MSP: Multimodal sensors (passive); MSA: Multimodal sensors (active); STT: Single target (true); STF: Single target (false); MT: Multiple target.

**Table 4:** Average center location error (in pixels).

Sequence	Algorithm			
	CT	BSBT	SemiT	Struck (GPU)
Panda (1000 fp)	69	80	70	**13**
Crowds (346 fp)	66	6	**5**	10
Sylvester (1343 fp)	52	76	71	**31**
Indoor1 (269 fp)	28	25	13	**7**
Indoor2 (239 fp)	40	29	15	**6**
Indoor3 (230 fp)	**9**	31	15	28
Harbor1 (172 fp)	37	8	10	**4**
Harbor2 (86 fp)	5	**3**	**3**	4
Harbor3 (147 fp)	19	3	5	**2**
Average FPS	34.7	4.5	5.1	**42**

The bolded figures mean better.

**Table 5:** Success rate (%).

Sequence	Algorithm			
	CT	BSBT	SemiT	Struck (GPU)
Panda (1000 fp)	19	25	30	**100**
Crowds (346 fp)	7	15	10	**77**
Sylvester (1343 fp)	32	50	49	**86**
Indoor1 (269 fp)	**100**	99	87	98
Indoor2 (239 fp)	96	81	95	**99**
Indoor3 (230 fp)	36	80	79	**99**
Harbor1 (172 fp)	19	54	51	**98**
Harbor2 (86 fp)	**100**	97	98	**100**
Harbor3 (147 fp)	28	98	97	**99**

The bolded figures mean better.

**Table 6:** Tracking success rates.

Source	Average of Single Sensor	Multimodal Sensors	Multimodal Sensors
		Passive	Active
Scene1 (True target only) (1247 fp)	72.3%	74.3%	**91.2%**
Scene2 (False target only) (1756 fp)	5.3%	17.6%	**43.5%**
Scene3 (Two Targets) (1357 fp)	35.2%	45.2%	**85.6%**
Scene4 (Two Targets) (1519 fp)	56.4%	59.4%	**84.2%**
Scene5 (Two Targets) (1823 fp)	45.8%	52.3%	**87.5%**
Average (Except Scene2)	52.43%	57.80%	**87.13%**
Total Average	43.00%	49.76%	**78.40%**

The bolded figures mean better.

## Abbreviations

The following abbreviations are used in this manuscript:

PTZ	Pan Tilt Zoom
LDV	laser Doppler vibrometer
RANSAC	RANdom SAmple Consensus
STT	Single target (true)
STF	Single target (false)
MSP	Multimodal sensors (passive)
MSA	Multimodal sensors (active)
MT	Multiple target
